# Going through the adolescence with disabled sibling: resilience as a protective factor for the occurrence of internalizing and externalizing disorders in adolescents

**DOI:** 10.1192/j.eurpsy.2024.1435

**Published:** 2024-08-27

**Authors:** M. Lipowska, A. Łada-Maśko, K. Lipowska, U. Sajewicz-Radtke, B. M. Radtke

**Affiliations:** ^1^Institute of Psychology, University of Gdańsk, Gdańsk, Poland; ^2^Department of Psychology, University of Amsterdam, Amsterdam, Netherlands; ^3^Laboratory of Psychological and Educational, Gdańsk, Poland

## Abstract

**Introduction:**

Presence of the disabled child in the family poses many challenges for their siblings, especially in adolescence. Children with disabled siblings often receive less attention from family and friends, experience a sense of injustice and anger towards sick siblings, as well as they are more likely to experience various somatic complaints and higher levels of depression and anxiety. However, research shows that resiliency could be a protective factor associated with the functioning of children and adolescents in certain life events, such as sibling’s disability, referring to good adaptation despite facing emerging adversities.

**Objectives:**

Therefore, the aim of the current study was to examine the role of resilience as a possible protective factor for the occurrence of internalizing and externalizing disorders in adolescents having disabled sibling.

**Methods:**

175 diads of a healthy adolescent and one of its parents (*N* = 350) participated in the study. Participants were divided into two groups - 119 diads in the group with disabled sibling (*M_adolescent’s age_* = 16.70; *SD* = .66) and 56 diads in the group with a healthy sibling (*M _adolescent’s age_* = 16.64; *SD* = .75). The following measures were used in the study: Resilience Measurement Scale (SPP-18) and Child Behavior Checklist for Ages 6-18 (CBCL/6-18).

**Results:**

The results showed no statistically significant differences in any of the measured resilience factors (optimistic attitude and energy, persistence and determination in action, sense of humor and openness to new experiences, personal competences and a tolerance for negative affect), in adolescents with disabled sibling, comparing to the control group. However, adolescents with disabled sibling were found to have a significantly higher risk of the occurrence of both, externalizing and internalizing disorders compared to adolescents with healthy siblings. Furthermore, findings of the study also confirmed that personal competences and a tolerance for negative affect predicts lower risk of the occurrence of internalizing disorders in adolescents having disabled sibling.

**Conclusions:**

Our findings highlights that resilience may have important role in reducing the risk of the clinical problems occurrence in adolescents having disabled sibling. Therefore, comprehensive psychological support enhancing their personal growth and competence should be provided in the above group.

**Disclosure of Interest:**

None Declared

